# Heterotypic Neuraminidase Antibodies Against Different A(H1N1) Strains are Elicited after Seasonal Influenza Vaccination

**DOI:** 10.3390/vaccines7010030

**Published:** 2019-03-13

**Authors:** Jose Manuel Mendez-Legaza, Raúl Ortiz de Lejarazu, Ivan Sanz

**Affiliations:** 1 Microbiology Service, Hospital Clínico Universitario de Valladolid, Avenida Ramón y Cajal s/n, 47005 Valladolid, Spain; rortizdelejarazu@saludcastillayleon.es (R.O.d.L.); isanzm@saludcastillayleon.es (I.S.); 2 Valladolid National Influenza Centre, Avenida Ramón y Cajal s/n, 47005 Valladolid, Spain

**Keywords:** neuraminidase, original antigenic sin, seasonal influenza vaccination

## Abstract

Neuraminidase (NA) content is not standardized in current seasonal influenza vaccines; neither anti-NA antibodies (anti-NA Abs) are measured nor is it well-defined as a correlate of humoral protection. In this work, the presence of NA1 antibodies against classical A(H1N1) and A(H1N1) pdm09 subtypes was studied before and after vaccination with seasonal vaccines containing A/California/07/2009 strain (A(H1N1) pdm09 subtype). By Enzyme-Linked Lectin Assay (ELLA; Consortium for the Standardization of Influenza Seroepidemiology), we analyzed serum samples from two different cohorts (adults and elderly). The presence of anti-NA Abs at titers ≥1/40 against classical A(H1N1) and A(H1N1) pdm09 subtypes were frequently found in both age groups, in 81.3% and 96.3% of adults and elderly, respectively. The higher titers of anti-NA Abs (NAI titers) were detected more frequently against classical A(H1N1) strains according to the expected age when the first flu infection takes place. In this way, an Original Antigenic Sin phenomenon related to NA seems to be part of the immune response against flu. Seasonal-vaccination induced homologous seroconversion against NA of A(H1N1) pdm09 subtype in 52.5% and 55.0%, and increased the Geometric Mean Titers (GMTs) in 70.0% and 78.8% of adults and elderly, respectively. Seasonal vaccination also induced a heterotypic anti-NA Abs response against classical A(H1N1) strains (seroconversion at least in 8.8% and 11.3% of adults and elderly, respectively, and an increase in GMTs of at least 28.0% in both age groups). These anti-NA Abs responses occur even though the seasonal vaccine does not contain a standardized amount of NA. This work demonstrates that seasonal vaccines containing the A(H1N1) pdm09 subtype induce a broad antibody response against NA1, that may be a target for future influenza vaccines. Our study is one of the first to analyze the presence of Abs against NA and the response mediated by NAI titers after seasonal influenza vaccination.

## 1. Introduction

Influenza infection is a major health problem in both developed and developing countries. The importance of influenza infection is due to both the high morbidity, which causes a significant number of people infected in a short period of time, and its related mortality either directly or by aggravating comorbidities. More than 80 years ago the first influenza virus was isolated [1] and since then there have been significant changes in the knowledge regarding influenza. 

Among influenza viruses, different strains of subtype A(H1N1) influenza have circulated in humans [2,3] until its extinction in 2009 coinciding with the emergence of the new pandemic strain, A(H1N1) pdm09 [4]. The first human infection with the influenza virus likely stimulates the production of key antibodies that then shape later immune responses to different seasonal influenza strains. The world’ population may have experienced different imprinting on its immune system. Individuals born before 1957 and those born before 2009 possibly show different immunological memory based on circulating antibodies against subtype A (H1N1) influenza.

Immune memory is the cornerstone of the practice of influenza vaccination. Vaccination programs are established in almost all developed countries with different population targets. In general, most countries emphasize the need for vaccination in those over 65 years of age and other risk groups such as individuals with comorbidities. There are numerous reports in the literature that have studied the humoral response measured by specific antibodies against hemagglutinin protein (HAI titers) as a correlate of protection and vaccine efficacy after seasonal vaccination [5,6]. In recent years, several studies have emphasized the doctrine of original antigenic sin regarding the efficiency and effectiveness of seasonal vaccination [7,8,9]. However, few studies have studied humoral response measured by specific antibodies against neuraminidase (anti-NA Abs), a first-line viral target with hemagglutinin for the clearance of influenza virus infection [10].

One of the limitations in current inactivated influenza vaccines is that its formulation is only standardized for hemagglutinin (HA) content and thus, humoral responses induced by these vaccines are related primarily to HAI titers [11,12]. However, it is of growing interest in influenza viral neuraminidase (NA) and in the role of a humoral response mediated by anti-NA Abs as a correlate of protection against influenza infection [13,14,15,16,17,18,19,20,21]. The threat of the influenza virus increases the preparedness of protective immunity to pandemic and seasonal infection by vaccination. Furthermore, a growing number of studies claim to include or increase the amount of NA in influenza vaccine composition in order to provide broader vaccine immunogenicity [22,23,24,25]. Several questions remain that need to be further addressed for the future development of innovative and rapidly efficient vaccines strategies.

One of the objectives of the Consortium for the Standardization of Influenza Seroepidemiology (CONSISE) is to promote the study of humoral response against other viral proteins beyond the HA, such as the NA [26]. As far as we know, this is one of the few recent European studies published and is the first study in Spain analyzing the presence of Abs against NA and the humoral response mediated by anti-NA Abs after seasonal influenza vaccination. Spain is one of the European countries with higher rates of vaccination, especially in those over 65 years of age [27], and contributes to a larger number of samples for epidemiological influenza surveillance conducted through Global Influenza Surveillance and Response System (GISRS) [28]. In addition, certain National Influenza Centers (NICs), such as the NIC Valladolid, have extensive experience in the annual collection of sera- and sero-epidemiological analysis, not only against subtypes and current strains but also against old influenza viruses [29,30].

In this work, we have studied the baseline level of anti-NA antibodies and the immune response mediated by anti-NA Abs against classical and recent influenza A (H1N1) and A(H1N1) pdm09 strains after the vaccination with A/California/07/2009 (A(H1N1) subtype pdm09) in adults and the elderly population.

## 2. Materials and Methods

### 2.1. Study Design

A retrospective experimental study was designed, analyzing the presence of anti-NA Abs in pre- and post-vaccination serum samples from 160 individuals. The study population was classified according to the age of the elderly (≥65 years; *n*_1_= 80) and adults (15–64 years; *n*_2_= 80) groups. These individuals were recruited during the 2013–2014 and 2014–2015 influenza vaccination campaigns (IVCs) [31,32]. The type of seasonal influenza vaccine administered in the adult group was a trivalent inactivated vaccine (*Vaxigrip*^®^, Sanofi Pasteur, Lyon, France) and an MF59-adjuvanted trivalent vaccine (*Chiromas*^®^, Novartis, Siena, Italy) was used in the elderly group, in both IVCs. These seasonal influenza vaccines included as a vaccine strain within subtype A(H1N1) to the strain A/California/07/2009 (subtype A(H1N1) pdm09) [31,32]. Pre-vaccination serum samples were obtained immediately prior to administration of the influenza vaccine and post-vaccine serum samples at least 28 days after its administration. Serological samples were obtained through physicians of the Sentinel Influenza Surveillance Network of Castile and Leon, Spain. Serological samples are sent annually to the National Influenza Center of Valladolid (Valladolid NIC) where these are analyzed. The collection of sera is regulated as part of a sero-epidemiological surveillance project through the Sentinel Influenza Surveillance Network of Castile and Leon (Spain), regulated through the Order SAN/1593/2006 of 13 October 2006. Informed consent was obtained and the recruitment of the Patients was done following the Spanish Organic Law for Data Protection, patient’s rights and Obligations for clinical documents (BOE no. 298 of 14 December 1999, Law 41/2002). This research was performed following the Declaration of Helsinki.

### 2.2. Influenza Viruses Analyzed

The presence of anti-NA Abs was analyzed against five different strains of seasonal subtype A(H1N1) and against one strain of subtype A(H1N1) pdm09. Strains were as follows: A/PuertoRico/8/1934 (A/PR/8/1934), A/Weiss/1943, A/FortMonmouth/1/1947 (A/FM/1/1947), A/Brazil/11/1978, A/Brisbane/59/2007, and A/California/07/2009 (A(H1N1) pdm09). These strains were selected for their relevance with regard to subtype A (H1N1) influenza evolution since its origin in 1918 [2] till its extinction in 2009 with the emergence of a new pandemic subtype, the A(H1N1) pdm09 [4]. A/PR/8/1934, A/Weiss/1943, and A/FM/1/1947 strains are three representative classical strains before the reemergence of subtype H1 in 1977 with the emergence of the A/USSR/90/77 strain [33]. These three strains were also the first H1 flu strains that were included in the first vaccines used in humans. After its reemergence in 1977 until its end in 2009, two important classic subtype H1 seasonal strains were A/Brazil/11/1978 and A/Brisbane/59/2007 strains. A/Brisbane/59/2007 is also the last strain related to seasonal subtype A (H1N1) selected as a vaccine strain before the extinction of this seasonal subtype. A/California/07/2009 (pdm09) is the first strain related to pandemic subtype A(H1N1) selected as a vaccine strain. A/PR/8/1934 was provided by the National Institute for Biological Standards and Controls (NIBSC, London, UK) which was previously inactivated with beta-propiolactone for its use in BSL-II conditions. Egg-adapted strains A/Weiss/1943, A/FM/1/1947, and A/Brazil/11/1978 were supplied by the Francis Crick Institute WHO Collaboration Center London (London, UK). A/Brisbane/59/2007 and A/California/7/2009 were obtained from the kit of reagents in the identification of influenza isolates provided by the International Reagent Resource (IRR), USA. Viral strains were grown in chicken embryos for 72 h at 35 °C in the NIC Valladolid for obtaining a sufficient viral load for experiments and then stored in aliquots at −80 °C until use. Embryos were supplied by Lohmann Breeders (Valladolid, Spain).

### 2.3. Immunological and Virological Assays

The analysis of the presence of specific anti-neuraminidase Abs (anti-NA Abs) in pre- and post-vaccine serum samples was performed by means of an enzyme-linked serological assay lectin (ELLA) [34,35,36]. Originally developed by Lambré [34], the ELLA assay is based on the release of terminal sialic acid residues from the fetuin which is used as a substrate. The advantages are that it evaluates specific NA antibodies, offers better safety and sensitivity, and requires no hazardous reagents. Assay repeatability as well as intra- and inter-laboratory variability was assessed in an international study of the ELLA, conducted through the Consortium for the Standardization of Influenza Sero-Epidemiology (CONSISE), and the results were quite promising [26]. In this research, we determined the magnitude and distribution of neuraminidase antibody responses against the historic and current influenza A(H1N1) and A(H1N1) pdm09 strains using the ELLA assay. As far as our knowledge, this is the first research in Spain that shows the results of human antibody response against neuraminidase. This practical method to measure anti-NA Abs titers (NAI titers) is performed in 96-well plates coated with a large glycoprotein substrate, fetuin. NA cleaves terminal sialic acids from fetuin, exposing the penultimate sugar, galactose. Peanut agglutinin (PNA) is a lectin with specificity for galactose and, therefore, the extent of desialylation can be quantified using a PNA-horseradish peroxidase conjugate, followed by addition of a chromogenic peroxidase substrate. The optical density measured is proportional to NA activity. First, a neuraminidase assay was performed to determine the optimal virus concentration to be used in the ELLA assay. This neuraminidase assay was performed for all viral stocks. To measure anti-NA Abs, serial dilutions of sera were incubated at 37 °C overnight (16–18 h) on fetuin-coated plates with a fixed amount of each virus. The reciprocal of the highest serum dilution that results in ≥50% inhibition of NA activity was designated as the NAI titer. The ELLA provides a practical format for the routine evaluation of human antibody responses following influenza infection or vaccination.

### 2.4. Phylogenetic Analysis

A phylogenetic analysis of the NA gene of the strains of subtype A(H1N1) and subtype A(H1N1) pdm09 was performed to study the phylogenetic relationships between those viruses. In that analysis, sequences of the complete NA gene of all strains included in the study were obtained from the database, Influenza Research Database (IRR), with the following accession numbers; A/PR/8/1934: 120393; A/Weiss/1943: 230628; A/FM/1/1947: 241812; A/Brazil/11/1978: 57807; A/Brisbane/59/2007: 502144; A/California/07/2009: 680484. The NA gene sequences were aligned using the ClustalW algorithm BioEdit 7.2.3. Phylogenetic analysis was performed using the MEGA 5.2 software (Mega Software, Tempe, AZ, USA) and the best nucleotide substitution model was predicted by the Best-Fit tool. The Hasegawa-Kishino-Yano model with gamma-distributed rates obtained the highest score Bayesian information criterion. The reproducibility of the phylogenetic tree was guaranteed by a bootstrap analysis of 1,000 replications. A genetic similarity matrix for the NA1 subunit was also constructed using the maximum-likelihood algorithm. The genetic similarity between the different influenza A(H1N1) and A(H1N1) pdm09 viruses were expressed as a percentage of genetic homology (% of similarity/100).

### 2.5. Statistical Analysis

In the statistical analysis, parameters such as the seroprotection rate (SPR), seroconversion rate (SCR), and the Geometric Mean Titers (GMTs) were calculated. Seroconversion was defined as an increase of at least four-fold titers between pre- and post-vaccine serum samples. Although there is currently a lack of consensus assuming the exact value of specific protective antibody titers against NA, in this work, we have used similar correlates of protection as those used for HAI titers [20]. Seroprotection analysis was assessed in duplicate considering NAI titers over 1/40 and over 1/80. The negative results obtained in NAI titers were assumed as half of the detection value (1/10) for the calculation of the GMTs. The statistical analysis was performed using different parametric and non-parametric methods as Student T test, Pearson chi-square test and McNemar by means of SPSS V24 software (IBM, Armonk, NY, USA). Statistical significance was taken at the *p* < 0.05 value.

## 3. Results

### 3.1. Population Characteristics

The mean age of the groups recruited for this study was 51.0 years (CI95%: 48.2-53.4) in the adult group and 78.6 years (CI95%: 76.6–80.6) in the elderly group. Distribution of groups mean age by influenza vaccine campaign (IVC) was: 50.3 years (CI95%: 46.5–54.2) and 76.0 years (CI95%: 73.9–78.1) in the 2013–2014 IVC; and 51.6 years (CI95%: 48.2–54.7) and 81.2 years (CI95%: 78.3–83.9) in 2014–2015 IVC, in adults and the elderly group, respectively. Men comprised 43.8% and 48.8% of those individuals recruited during the 2013–2014 and 2014–2015 influenza vaccine campaigns, respectively.

### 3.2. Presence of Pre-Vaccine Anti-NA Abs Against A(H1N1) and A(H1N1) pdm09

**Figure 1:**
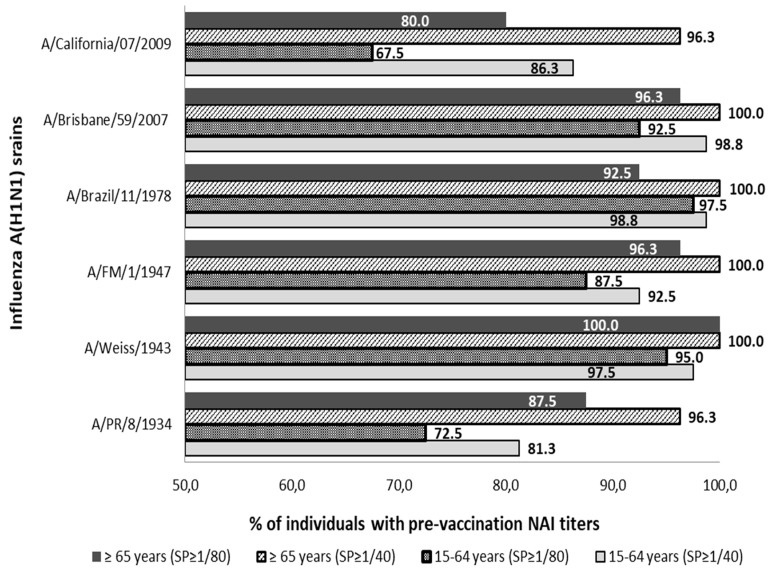
Percentage distribution of pre-vaccine sera with titers of anti-NA Abs (NAI titers) ≥1/40 and ≥1/80 against influenza strains of subtype A(H1N1) and subtype (H1N1) pdm09. SP; cut-off point NAI titers.

The presence of pre-vaccine Abs anti-NA was detected against all strains of subtype A(H1N1) and A(H1N1) pdm09 analyzed in both age groups. Pre-vaccine sera with titers ≥1/40 and ≥1/80 were observed in more than 81.3% and 67.5% of individuals in both population groups analyzed, see Figure 1.

All individuals in the age group ≥65 years showed NAI titers ≥1/40 against strains A/Weiss/1943, A/FM/1/1947, A/Brazil/11/1978, and A/Brisbane/59/2007. The lowest percentage of individuals ≥65 years with Abs ≥1/40 was observed against strains A/PR/8/1934 and A/California/07/2009 (*n* = 77; 96.3%) in both cases. On the other hand, no virus against which all individuals aged 15–64 showed antibody titers ≥1/40 was found. The highest percentage of adult individuals with titers of ≥1/40 was against strains A/Brazil/11/1978 and A/Brisbane/59/2007 (*n* = 79; 98.8%) in both cases, and the percentage was lower against the strain A/PR/8/1934 (*n* = 65; 81.3%).

Analyzing both groups with the cut-off point for Abs titer in ≥1/80, it was observed that all individuals of group ≥65 years showed titers ≥1/80 against strain A/Weiss/1943. The lowest percentage of individuals ≥65 years with Abs to ≥1/80 was observed against the strain A/California/07/2009 (*n* = 64; 80.0%). On the other hand, no virus was found against which all individuals aged 15–64 showed antibody titers ≥1/80. The highest percentage of adult individuals with titers of ≥1/80 was against the strain A/Brazil/11/1978 (*n* = 78; 97.5%) and the lowest rate was against the strain A/California/07/2009 (*n* = 54; 67.5%).

The percentage of individuals with titers ≥1/40 was significantly higher in the elderly group than in the adult group against strains A/PR/8/1934, A/FM/1/1947 and A/California/07/2009 (Pearson chi-square; *p* < 0.05). These differences were also significantly higher in the elderly even when a titer of ≥1/80 was considered against strains A/PR/8/1934, A/Weiss/1943 and A/FM/1/1947 (Pearson chi-square; *p* < 0.05).

The highest value of pre-vaccination GMTs in individuals ≥65 years was observed against strain A/Weiss/1943 (GMTs = 716.0; CI95%: 534.0–974.7) and lowest against the strain A/California/07/2009 (GMTs = 136.9; CI95%: 109.2–170.9) (Table 1). In the aged 15–64 years group, the highest GMTs was observed against strain A/Brazil/11/1978 (GMTs = 448.5; CI95%: 364.5–542.9) and lowest against A/California/07/2009 (GMTs = 100.2; CI95%: 79.3–127.7). GMTs was significantly higher in the elderly group against the A/PR/8/1934, A/Weiss/1943 and A/FM/1/1947 and significantly higher in adults than in elderly group against the strain A/Brazil/11/1978 (Student-T; *p* < 0.05). Significant differences in GMTs between both age groups against strains A/Brisbane/59/2007 and A/California/07/2009 were not found.

### 3.3. Heterotypic Anti-NA Abs Response Induced by Influenza Seasonal Vaccination

Seasonal vaccination with the strain A/California/07/2009 (subtype A(H1N1) pdm09) induced a significant heterotypic anti-NA Abs response against all A(H1N1) subtype strains analyzed in both age groups (McNemar; *p* < 0.05). The highest heterologous seroconversion rates (SCR) were observed in the adult group against the strain A/Weiss/1943 (SCR = 31.3%) and against the strain A/Brisbane/59/2007 in the ≥65 years (SCR = 36.3%), see Table 2. The seroconversion rate was significantly higher in the elderly than in adults against the strain A/Brisbane/59/2007 and also significantly higher in adults than the elderly against the strain A/Weiss/1943 (Pearson chi-square; *p* < 0.05). Seasonal vaccination produced a homologous seroconversion against the strain included in the vaccine composition (A/California/07/2009) in 52.5% of adults (*n*_1_ = 42) and 55% of individuals ≥65 years (*n*_2_ = 44). No significant differences in SCR between both age groups against this strain were found (Pearson chi-square; *p* < 0.05).

**Figure 2:**
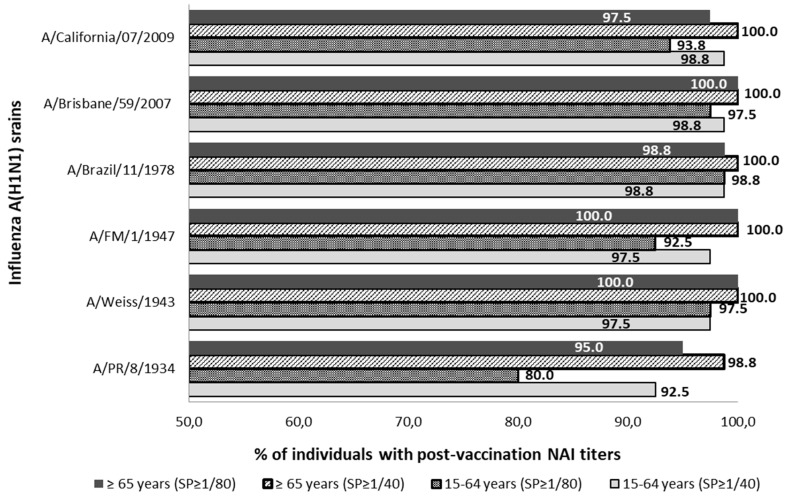
Percentage distribution of post-vaccine sera with NAI titers ≥1/40 and ≥1/80 against influenza strains of subtype A(H1N1) and subtype A(H1N1) pdm09. SP; cut-off point NAI titers.

The percentual distribution of individuals with NAI titers ≥1/40 and ≥1/80 after seasonal vaccination is described in Figure 2. After vaccination, the SPR ≥1/40 reached was over 92.5% and over 98.8% against all influenza strains studied in the adult and elderly groups, respectively. Considering those NAI titers over 1/80, the SPR ≥ 1/80 reached was over 80.0% and over 95.0% against all influenza strains studied in the adult and elderly groups, respectively. Statistically significant differences between the SPR ≥ 1/40 prior to and after vaccination were observed in the young-adult group against strains A/PR/8/1934 and A/California/07/2009 and in the elderly group against A/PR/1934 and A/California/2009 strains (McNemar; *p* < 0.05).

**Figure 3:**
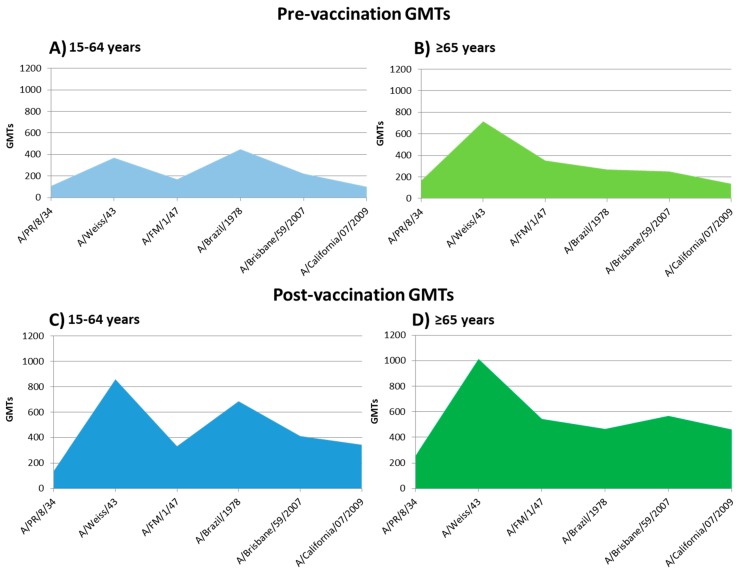
Profile of pre-vaccine GMTs and its increase after seasonal influenza vaccination. (**A**): pre-vaccine GMTs of 15–64 years group; (**B**): pre-vaccine GMTs of the elderly group; (**C**): post-vaccine GMTs of 15–64 years group; (**D**): post-vaccine GMTs of the elderly group.

Post-vaccination GMTs against the influenza A(H1N1) strains analyzed are described in Table 3. Post-vaccine GMTs observed were significantly higher against strains A/PR/8/1934, A/FM/1/1947, and A/Brisbane/59/2007 in the elderly rather than in the adult group. On the other hand, these post-vaccine GMTs were higher against the strain A/Brazil/11/1978 in adults than in the elderly group (Student-T; *p* < 0.05). The profile of pre-vaccine GMTs and its increase after seasonal influenza vaccination are shown in Figure 3.

### 3.4. Phylogenetic Analysis of Influenza Viruses Studied

**Figure 4:**
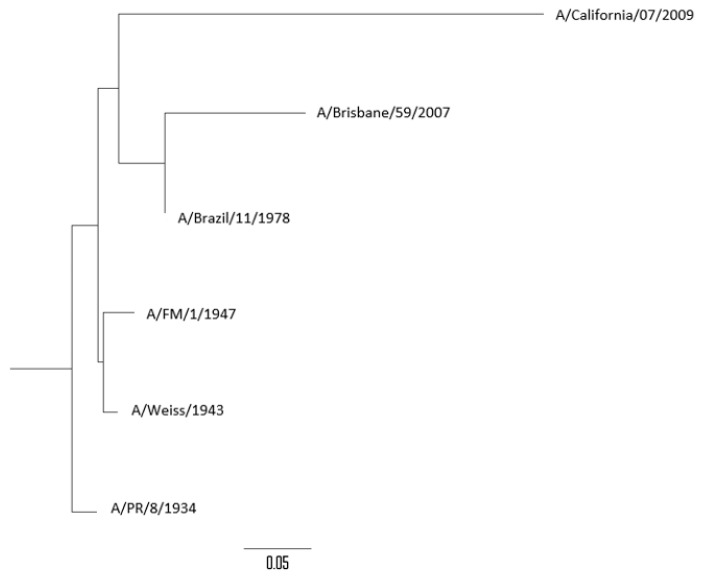
Phylogenetic tree of the neuraminidase (NA) gene of influenza A(H1N1) strains analyzed.

The percentages of genetic similarity (% of similarity/100) of the NA gene between the different influenza A(H1N1) and A(H1N1) pdm09 viruses analyzed are described in Table 4. Genetic homology of the NA gene observed between the different strains studied was over 85%. The highest genetic homology observed was between A/Weiss/1943 and A/FM/1/1947 strains (96.7%) and the lowest was the genetic homology observed between A/PR/8/1934 and A/Brisbane/59/2007 strains (85.6%). The pandemic strain A/California/07/2009 (subtype A(H1N1) pdm09) showed a genetic homology with seasonal strains (H1N1) between 72.1% (A/Brisbane/59/2007) and 76.4% (A/FM/1/1947). The phylogenetic tree constructed by using the NA gene of all viruses analyzed is shown in Figure 4.

## 4. Discussion

The efficacy of serological protection against influenza viruses has traditionally been related to the production of neutralizing Abs specifically against the hemagglutinin (HA). The immunogenicity of currently licensed influenza vaccines is focused on the content of the HA antigen, without giving greater importance to the content of other viral proteins. Purification methods of these vaccines are intended to preserve the HA structure but not the NA. Because of this, the total amount of NA in these vaccines is unknown and is not standardized [12]. Currently licensed influenza vaccines contain purified and standardized HA but also contain residual NA antigen [37,38,39].

The humoral responses after seasonal vaccination measured as anti-HA Abs production (hemagglutination inhibition [HAI]) are widely described in scientific literature, but less is known about the humoral response to the NA induced by these vaccines. There are published works that study the role of anti-NA Abs for the protection against influenza viruses in animal and human studies [20,23,40,41]. However, there are few studies that analyze the NA antibody responses provided by the current seasonal influenza vaccination in different human populations [42]. As far as our knowledge, this is the first research in Spain that studies the anti-NA Abs response prior to and after vaccination with seasonal influenza vaccines. 

Our data demonstrate the existence of a high percentage of adults and elderly people showing Abs against NA1 neuraminidase of the influenza A(H1N1) and A(H1N1) pdm09 analyzed strains. The elderly people were the age group with the highest seroprotection rate against those viruses. The demonstration of the presence of these anti-NA Abs is relevant. Despite the fact that the origin of these Abs is unknown in both age groups, it can be as a result of two different causes. These Abs could have been induced by past influenza infections by different A(H1N1) subtype strains, and our results would demonstrate that immune memory also happens for the NA protein [18,21]. However, these Abs could be induced by previous vaccinations with related influenza strains that have generated heterotypic responses against all the strains analyzed.

Pre-vaccine GMTs were significantly higher in the elderly than in adults against A/PR/8/1934, A/Weiss/1943, and A/FM/1/1947 strains. On the other hand, GMTs were significantly higher in adults than the elderly for A/Brazil/11/1978 strain. These differences can be caused by the first contacts of these age groups against influenza. Taking the age of the elderly people analyzed, they were probably primed by viruses that circulated during the 1930s or 1940s, which were descendent from the Spanish Influenza. On the other hand, adults were probably primed by A(H1N1) viruses that circulated after their re-emergence in 1977. In this context, the first time that a person comes into contact with an influenza virus can be crucial and can condition the following interactions with other influenza viruses. This phenomenon is known as the “Doctrine of original antigenic sin” and has been described for the humoral response to the influenza hemagglutinin [7,8,9], but not for the neuraminidase. The data from our study show that this interesting effect also occurs against NA. As far as we know, this is the first work that demonstrates this particularity for this protein. In short, our study represents a reflection of group immunity correlated with age and with an individual’s first influenza infection in their life, demonstrating that the Original Antigenic Sin is also closely related to the humoral response to NA. Further research is needed to compare HA with NA when the original antigenic sin in the influenza virus is mentioned.

The results of our study demonstrate that seasonal vaccination against the A/California/07/2009 strain (A(H1N1) pdm09 subtype) induced significant homologous and heterologous humoral responses to NA1 against all A(H1N1) and A(H1N1) pdm09 strains analyzed in this work, both in adults and in the elderly. This response occurs despite the fact that the vaccine does not have a standardized amount of NA in its composition [37,38,39].

Seasonal vaccination induced homologous seroconversion against NA of subtype A(H1N1) pdm09 in more than half of the individuals in both age groups. Influenza vaccination also induced heterotypic seroconversion in NAI titers against A(H1N1) strains analyzed not included in the seasonal vaccine. This heterotypic anti-NA Abs response may be due to the high genetic homology between the NA protein of both A(H1N1) and A(H1N1) pdm09 influenza subtypes (more than 70%). The NA protein is present at the surface of the influenza virus and shows a lower mutation rate than HA [10,43]. Thus, the NA protein can remain without antigenic changes for longer times than HA. This lower variability of NA allows the existence of conserved epitopes in NAs proteins of the same clade [44], favoring the heterotypic reactions. Because of this, the results of our study not only show that seasonal influenza vaccines against the A(H1N1) pdm09 subtype induced a humoral response against a protein that is not standardized in their composition, the NA, but also induced interesting heterotypic responses. This emphasizes the importance of the design of future influenza vaccines based on the NA protein or standardizing their content in the currently used seasonal vaccines. Since the main objectives of the design of future influenza vaccines are an increase in the heterotypic responses (universal vaccine) and also include new viral targets for inducing wider humoral responses, these results show that NA is one of the most promising targets to address these requirements. Further research is needed to determine how these influenza vaccines could induce heterotypic humoral protection against other viruses containing NA1 neuraminidase, like A(H5N1), as it has been described against viral hemagglutinin [29].

There are certain circumstances in which the response to vaccines may turn out to be lower than expected, as in the case of the elderly population with the immune senescence [45,46,47,48]. However, compared to adults, the immune senescence seems to not be appreciated in our study. The seroconversion after vaccination was similar in both age groups with no significant differences for most of the viruses analyzed. However, we observed statistically significant differences in the SCR after vaccination against the A/Weiss/1943 strain, being higher in the adults. This is surprising because adults have never been in contact with these viruses since they have born after 1960. This issue is probably a bias caused by the high pre-vaccine NAI titers of the elderly, which could have hindered the NAI titers increasing as much as in adults, which showed lower pre-vaccine GMTs. These results seem to show that the conserved structure of NA could have been mitigating the effect of immune senescence, which can be interesting for influenza vaccination. 

The GMTs achieved after vaccination doubled in many cases that which was observed before vaccination against almost all strains in both groups. This demonstrates the ability of a seasonal influenza vaccine to raise the Abs titers against NA, an aspect that we believe should be taken into account for the design of future influenza vaccines. 

The results of our study show that seasonal influenza vaccines (SIV) increase NAI titers in a large percentage of the population analyzed despite the fact that SIV does not include a standardized content of NA in its composition. The implication of this anti-NA Abs response in terms of serological protection against the influenza virus cannot be evaluated using the methodology used, so it is necessary to conduct studies in vivo to determine the effectiveness of a hypothetical influenza vaccine that contains a standardized amount of NA. Previous studies have evaluated the presence of anti-NA Abs and their relationship to immunological protection against influenza, both in animals and humans exposed to the influenza viruses [20,23,40,41]. Our data and data from previous studies support that humoral immunity based on the NA plays an important role in the protection against flu and that the NA antigen should be taken into account for the design of future influenza vaccines.

Among the limitations of this study, it should be noted that complete viruses have been used with native forms of HA and NA from human seasonal A(H1N1) and A(H1N1) pdm09 strains. According to some authors, the ELLA assay can be interfered by preexisting antibodies against HA through different mechanisms of steric hindrance between the anti-HA antibodies and the NA [49], artificially increasing NAI titers. Because of this, some results of our study may overestimate NAI titers by the possible presence of Abs against H1 [36]. However, the use of chimeric viruses containing exotic haemagglutinin as H6Nx for the evaluation of anti-NA Abs may also be limited by the presence of heterotypic antibodies in the analyzed population. Our group has previously demonstrated that a moderate percentage of the population ≥65 years have heterotypic antibodies at protective titers against the globular head of the HA of non-seasonal influenza viruses such as H5 and H9 [29]. These antibodies were present as a result of imprinting with seasonal influenza viruses, as H1 and H2, during their life. These heterotypic antibodies have also been observed by other authors [50]. H5 and H9 subtypes belong to clade 1 of the HA and present similar or even less genetic homology than the H6 subtype with respect to human seasonal influenza viruses H1 and H2. Given the fact that these heterotypic antibodies are commonly found in exposed and vaccinated populations, it is difficult to evaluate the extent of the interaction of these heterotypic antibodies in the ELLA assay when using exotic and native hemagglutinins. 

Despite the fact that HA antibodies could be artificially increasing titers of the anti-NA antibodies in this study by steric hindrance, we think that this effect is limited. In this regard, it is worth commenting on some aspects of the architecture of the envelope of influenza viruses in relation to HA and NA. Firstly, some authors suggest that the neutralization ability of the stem-specific anti-HA antibodies could be limited by the disposition of the HA on the surface of the influenza virus, due to their limited access to the membrane-proximal epitopes [51,52]. Also, the immune subdominance of the HA stem could affect the production of specific antibodies against this region [53]. While some authors demonstrated by computational methods that the HA structure in the virion surface does not limit the access of the stem-specific antibodies to the membrane-proximal epitopes [54], the immune subdominance of the stem region of the HA implies a lower production and variety of antibodies with respect to the HA globular head. This issue also affects the steric hindrance between HA and NA, since this effect can be mitigated by the limited number and variety of these antibodies. Secondly, the arrangement of HA and NA on the surface of the virion occurs in the form of patches, in which several proteins of the same class are arranged (HA on one side and NAs on others). In these patches, there are several NAs together and only those that are part of the outside of the patch are those that can suffer the steric hindrance effect with the HAs that are immediately close to NA. In fact, in some cases, even the NAs are situated near to an ion channel or M2 protein. Because of this, the steric hindrance effect produced by the anti-HA antibodies present in the serum would only occur against the NAs situated outside of these patches.

Also, in this work, we have used two cut-off points assimilating NAI titers over 1/40 and over 1/80 due to lack of consensus assuming the exact value on the seroprotection against NA. There are some studies that have determined equivalent seroprotection correlates between both proteins [18,19,20], so the results of this study provide information on this type of comparison to the studies already available. The analysis of the antibodies against NA in a human model prior to vaccination and those induced by the seasonal vaccine in this study offer an interesting point of view on how the humoral immune response happens in the reality after vaccination. The publication of new studies such as this will allow for the comparison and analysis of the anti-NA Abs response in terms of humoral protection and vaccine serological efficacy.

## 5. Conclusions

In summary, the presence of Abs at protective titers against the NA of A(H1N1) and A(H1N1) pdm09 subtypes were frequently found in both populations studied, adults and the elderly. The higher titers of anti-NA Abs were detected more frequently against classical influenza A(H1N1) strains according to the age of the first flu infection. In this way, an Original Antigenic Sin related to NA seems to be present. Although seasonal influenza vaccines do not contain a standardized amount of NA, at least 28% of vaccinated adults and elderly showed increased NAI titers. Seasonal vaccination containing A/California/07/2009 strain (A(H1N1) pdm09) induced homologous anti-NA Abs response against A(H1N1) pdm09 subtype but also heterologous responses against classical strains of A(H1N1) subtype. Knowledge of these responses to vaccination can contribute to the design of new influenza vaccines that expand the protection against proteins that are non-standardized in seasonal influenza vaccines. The results of our study demonstrate that NA antigen could be included in the Next Generation Influenza Vaccines. Further research is needed to understand the link of NAI titers and its correlation with HAI titers in protection, as well as the optimal dose of NA antigen to generate that response.

## Figures and Tables

**Table 1:** Geometric Mean Titers (GMTs) of anti-NA antibodies in both age groups against A(H1N1) and A(H1N1) pdm09 strains. Comparison between GMTs of the adult and elderly groups. (CI: confidence intervals).

A(H1N1) and A(H1N1) pdm09 Strains	Anti-NA GMTs (CI 95%)		*p*-Value
	Adults (15–64 Years)	Elderly (≥65 Years)	
A/PR/8/1934	106.5 (81.2–137.7)	166.6 (131.1–209.9)	0.016
A/Weiss/1943	369.6 (269.9–485.7)	716.0 (534.0–974.7)	0.002
A/FM/1/1947	169.5 (128.3–215.5)	351.9 (283.9–434.4)	<0.001
A/Brazil/11/1978	448.5 (364.5–542.9)	269.0 (211.8–336.1)	0.001
A/Brisbane/59/2007	222.3 (179.5–276.4)	251.0 (211.4–296.5)	0.399
A/California/07/2009	100.2 (79.3–127.7)	136.9 (109.2–170.9)	0.066

**Table 2:** Number of seroconversions and seroconversion rate against classical influenza A(H1N1) strains and A(H1N1) pdm09 strain after seasonal flu vaccination.

A(H1N1) and A(H1N1) pdm09 Strains	Adults (15–64 Years)		Elderly (≥65 Years)	
	SCN	SCR	SCN	SCR
A/PR/8/1934	7	8.8	9	11.3
A/Weiss/1943	25	31.3	11	13.8
A/FM/1/1947	17	21.3	10	12.5
A/Brazil/11/1978	12	15.0	15	18.8
A/Brisbane/59/2007	14	17.5	29	36.3
A/California/07/2009	42	52.5	44	55.0

SCN: Number of seroconversions; SCR: Seroconversion rate.

**Table 3:** Geometric mean titers (GMTs) of anti-NA Abs against different influenza A(H1N1) strains after seasonal flu vaccination.

A(H1N1) and A(H1N1) pdm09 Strains	Anti-NA GMTs (CI 95%) Post-vaccination		*p*-Value
	(15–64 Years)	(≥65 Years)	
A/PR/8/1934	139.3 (109.3–176.8)	253.2 (202.6–315.1)	<0.001
A/Weiss/1943	858.9 (639.7–1115.6)	1,012.5 (772.2–1349.4)	0.402
A/FM/1/1947	331.2 (251.8–426.1)	542.6 (450.7–656.2)	0.002
A/Brazil/11/1978	685.6 (533.6–861.9)	464.3 (378.8–583.3)	0.012
A/Brisbane/59/2007	411.3 (326.5–515.2)	566.7 (471.4–677.2)	0.028
A/California/07/2009	342.8 (270.2–435.3)	460.3 (370.3–563.6)	0.077

**Table 4:** Genetic similarity expressed as a percentage of similarity/100 of the NA1 subunit of the neuraminidase gene between the different influenza A(H1N1) and A(H1N1) pdm09 strains analyzed.

A(H1N1) and A(H1N1) pdm09 Strains	A/California/ 07/2009	A/Brisbane/ 59/2007	A/Brazil/ 11/1978	A/FM/ 1/1947	A/Weiss/ 1943	A/PR/8/ 1934
A/California/07/2009	1.000	-	-	-	-	-
A/Brisbane/59/2007	0.721	1.000	-	-	-	-
A/Brazil/11/1978	0.762	0.909	1.000	-	-	-
A/Weiss/1943	0.764	0.857	0.929	1.000	-	-
A/Brisbane/59/2007	0.752	0.864	0.941	0.967	1.000	-
A/PR/8/1934	0.753	0.856	0.920	0.938	0.950	1.000
